# CDK Inhibitors and FDA: Approved and Orphan

**DOI:** 10.3390/cancers16081555

**Published:** 2024-04-19

**Authors:** Jonas Cicenas, Jokubas Simkus

**Affiliations:** 1 MAP Kinase Resource, Bioinformatics, Melchiorstrasse 9, CH-3027 Bern, Switzerland; jo.simkus@gmail.com; 2 Secondary School “Varnų sala”, Baltupio g. 14, LT-08304 Vilnius, Lithuania; 3 Faculty of Medicine, Vilnius University, LT-01513 Vilnius, Lithuania

The protein kinases are a large family of enzymes which catalyze protein phosphorylation at certain amino acids. There are two large subfamilies of protein kinases: the tyrosine kinases and the serine/threonine kinases. The human genome contains 518 protein kinases, 478 of which contain a typical protein kinase domain, while 40 contain atypical protein kinases [1]. Protein phosphorylation by kinases plays a significant role in the regulation of numerous cell processes, such as proliferation, cell growth, the cell cycle, cell differentiation, cell survival and apoptosis [2,3]. The aberrant regulation of kinases can lead to notable changes in these processes and can be central for various diseases, such as cancers, neurodegenerative diseases, inflammation, diabetes, kidney diseases and cardiovascular disorders [2,4]. Cyclin-dependent kinases (CDKs) are a subgroup of protein kinases which play a crucial role in the control of the cell cycle, proliferation, transcription and gene expression [5]. There are 21 genes that encode CDKs in the human genome, as well as 5 genes that encode similar protein kinases dubbed as CDK-like kinases (CDKLs) [6].

Small-molecule kinase inhibitors are gaining increasing importance in the field of drug research, development and use. There are many such inhibitors, some of which are more specific while some are less, and quite a few of them have already been approved for cancer therapy [7], or are at least in clinical studies of different phases [8,9,10]. CDK inhibitors are no exception, and there are many inhibitors used for research purposes and for therapeutic applications. Many CDK inhibitors have been enrolled in various phases of clinical trials both for cancers and other diseases [11,12,13]. This editorial will provide an overview of the inhibitors which are approved by U.S. Food and Drug Administration (FDA) for the treatment of various diseases, as well as those which are granted the designation of orphan drugs.

Palbociclib (PD0332991, Ibrance) (Figure 1) is a specific CDK4/6 inhibitor. It has a potency against cyclin D1/CDK4 (IC50 = 11 nM) and cyclin D1, 2, 3/CDK6 (IC50 = 16 nM), but almost none against other CDKs (IC50 > 10,000) [14]. In 2012, it was predicted that PD0332991 would be the first approved CDK inhibitor [15]. On 3 February 2015, the FDA granted accelerated approval to palbociclib for the treatment of postmenopausal patients with estrogen receptor-positive, HER2-negative metastatic breast cancer. It is to be used in combination with letrozole as an initial endocrine-based therapy for women with metastatic disease. Its approval was based on a randomized, multicenter, open-label phase I/II trial, PALOMA-1 [16], which was confirmed by PALOMA-2 [17] and PALOMA-4 [18] phase III trials. On 19 February 2016, the FDA approved palbociclib for use in combination with fulvestrant for the treatment of women with hormone receptor-positive, HER2-negative advanced or metastatic breast cancer with disease progression following endocrine therapy. This approval was based on a randomized, multicenter, open-label phase III trial, PALOMA-3 [19]. On 4 April 2019, the FDA approved a supplemental new drug application for palbociclib for male patients with hormone receptor-positive, HER2-negative advanced or metastatic breast cancer. This approval was primarily based on the results of the PALOMA-2 and PALOMA-3 trials and supported by data from two phase I studies with palbociclib in males [20].

Ribociclib (LEE011, KISQALI) (Figure 1) has slightly greater potency against cyclin D1/CDK4 (IC50 = 10 nM) than against cyclin D1, 2, 3/CDK6 (IC50 = 39 nM), but almost none against other CDKs (IC50 > 50,000) [21]. On 13 March 2017, the FDA approved ribociclib in combination with an aromatase inhibitor as an initial endocrine-based therapy for the treatment of postmenopausal women with hormone receptor-positive, HER2-negative advanced or metastatic breast cancer. This approval was based on a randomized, double-blind, placebo-controlled, international clinical trial MONALEESA-2 [22], which was confirmed by MONALEESA-3 [23,24,25] and MONALEESA-7 [26,27] phase III trials. On 10 December 2021, the FDA approved ribociclib in combination with an aromatase inhibitor as an initial endocrine-based therapy in adult patients, or with fulvestrant as an initial endocrine-based therapy or following disease progression on endocrine therapy, in postmenopausal women and men. This approval was primarily based on the results of the MONALEESA-2 and MONALEESA-7 trials and supported by COMPLEEMENT-1 in males [28].

Abemaciclib (LY2835219, Verzenio) (Figure 1) has slightly greater potency against cyclin D1/CDK4 (IC50 = 2 nM) than against cyclin D1, 2, 3/CDK6 (IC50 = 10 nM), but significantly less against other CDKs (IC50 > 300) [29]. On 28 September 2017, the FDA approved abemaciclib in combination with fulvestrant for women with hormone receptor-positive, HER2-negative advanced or metastatic breast cancer with disease progression following endocrine therapy. This approval was based on MONARCH 2, a randomized, placebo-controlled, multicenter trial [30]. On 12 October 2021, the FDA approved abemaciclib with endocrine therapy (tamoxifen or an aromatase inhibitor) for the adjuvant treatment of adult patients with hormone receptor-positive, HER2-negative, node-positive early breast cancer at a high risk of recurrence. An additional requirement was having a Ki-67 score of greater than or equal to 20%. This approval was based on the monarchE trial [31,32]. On 3 March 2023, the FDA approved abemaciclib with endocrine therapy (tamoxifen or an aromatase inhibitor) for the adjuvant treatment of adult patients with hormone receptor-positive, human epidermal growth factor receptor 2-negative, node-positive early breast cancer at a high risk of recurrence. This approval removed the Ki-67 testing requirement [33].

SLS009 (GFH009) (Figure 2) is a specific CDK9 inhibitor. It has a potency against cyclin T1/CDK9 (IC50 = 9 nM), but almost none against other CDKs (IC50 > 100 at least) [34]. On 11 October 2023, the FDA granted an orphan drug designation to SLS009 for the treatment of patients with acute myeloid leukemia [35]. This was based on an open-label, single-arm, multicenter phase 1/2a trial (NCT04588922) in patients with relapsed/refractory AML and other hematologic malignancies [36]. On 30 October 2023, the FDA granted a fast-track designation for the treatment of relapsed or refractory peripheral T-cell lymphoma [37].

TP-1287 is a prodrug of alvocidib (Flavopiridol, L-868275, HMR-1275, NSC-649890) (Figure 2), which is a broad-range CDK inhibitor. It inhibits CDK1, CDK2, CDK9 (IC50 = 5–15 nM), CDK4, and CDK6 (IC50 = 40–300 nM) [13,38]. On 10 April 2023, the FDA granted an orphan drug designation to TP-1287 for the treatment of patients with Ewing sarcoma [39]. The FDA has also granted an orphan drug designation to alvocidib for the treatment of patients with acute myeloid leukemia [40].

NUV-422 (Figure 2) is an inhibitor of CDK2, CDK4 and CDK6. On 11 March 2021, the FDA granted an orphan drug designation to NUV-422 for the treatment of patients with malignant glioma [41]. This decision was based on a phase 1/2 study of NUV-422 (NCT04541225) in patients with high-grade gliomas [42]. However, this study was put on hold and later terminated due to the development of uveitis in patients [43]. The company eventually decided to discontinue the development of clinical development [44]. All of the inhibitor information mentioned in this editorial is summarized in Table 1.

The successes and failures of CDK inhibitors show us that this chapter of targeted therapy is far from its conclusion. One way or another, we have 21 CDK genes in the human genome as well as 5 similar kinases, and a only couple of them have clinically approved inhibitors and/or are close to reaching this goal. Both broad-spectrum CDK inhibitors as well as very selective ones, or ones which are selective for a small group of CDKs, should still be considered. A lot of clinical and preclinical research has been performed on previously developed small-molecule inhibitors, as well as new approaches [45]. In addition, there is always a possibility to develop alternative modes of inhibition, such as peptides or aptamers.

## Figures and Tables

**Figure 1:**
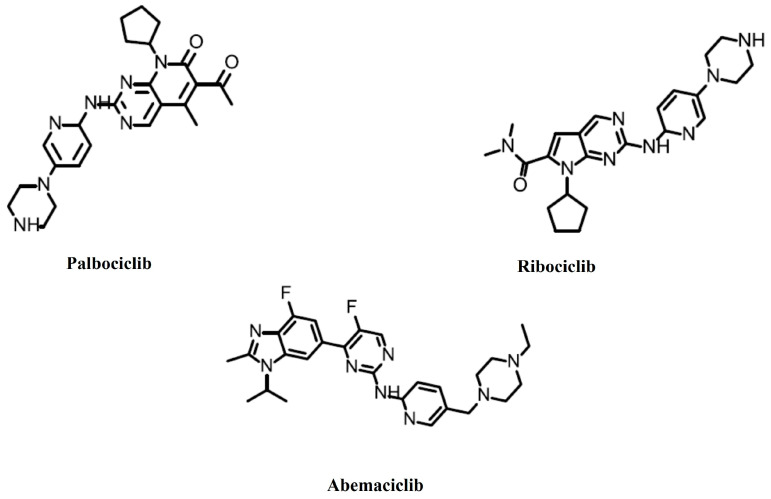
Food and Drug Administration-approved CDK4/6 inhibitors.

**Figure 2:**
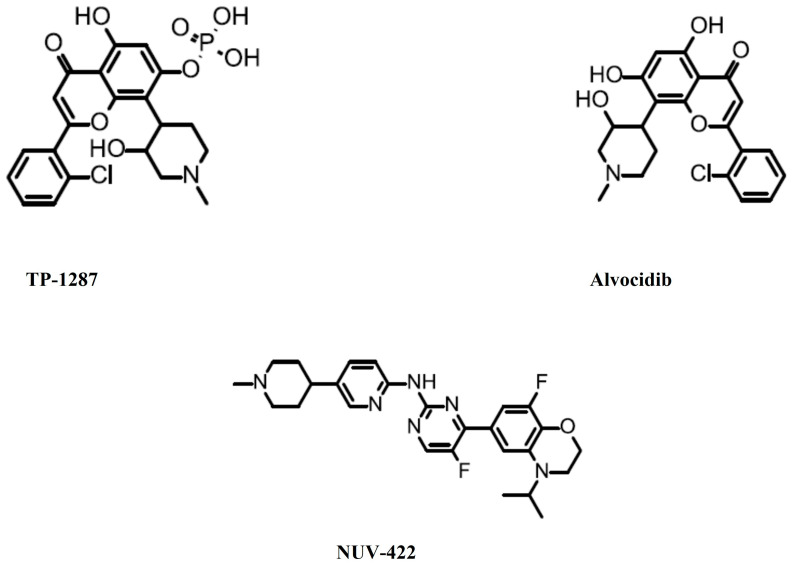
Inhibitors granted an orphan drug designation by the Food and Drug Administration.

**Table 1:** Summary of all CDK inhibitors mentioned in this editorial.

Inhibitor	CDKs	Diseases	References
Palbiciclib	CDK4/6	Metastatic breast cancer	[16,17,18,19,20]
Rocociclib	CDK4/6	Metastatic breast cancer	[22,23,24,25,26,27,28]
Abemaciclib	CDK4/6	High risk early breast cancer	[30,31,32,33]
SLS009	CDK9	Acute myeloid leukemia, peripheral T-cell lymphoma	[36,37]
TP-1287	Broad-range	Ewig sarcoma	[39]
Alvocidib	Broad-range	Acute myeloid leukemia	[40]
NUV-422	CDK2/4/6	Malignant glioma (discontinued)	[41,42,43,44]
